# APIO-EE-9 is a novel Aurora A and B antagonist that suppresses esophageal cancer growth in a PDX mouse model

**DOI:** 10.18632/oncotarget.18508

**Published:** 2017-06-16

**Authors:** Guoguo Jin, Ke Yao, Zhiping Guo, Zhenjiang Zhao, Kangdong Liu, Fangfang Liu, Hanyong Chen, Dhilli Rao Gorja, Kanamata Reddy, Ann M. Bode, Ziming Dong, Zigang Dong

**Affiliations:** ^1^ The Hormel Institute, University of Minnesota, Austin, Minnesota, USA; ^2^ Pathophysiology Department, School of Basic Medical Sciences, Zhengzhou University, Zhengzhou, Henan, P.R. China; ^3^ China-US (Henan) Hormel Cancer Institute, Zhengzhou, Henan, P.R. China; ^4^ The Henan Luoyang Orthopedic Hospital, Zhengzhou, Henan, P.R. China

**Keywords:** targeted therapy, aurora kinases, mitosis, esophageal cancer

## Abstract

Esophageal cancer (EC) is one of the most aggressive malignancies of the upper aerodigestive tract. Over the past three decades, with advances in surgical techniques and treatment, the prognosis of esophageal cancer has only slowly improved. Thus identifying novel molecular targets and developing therapeutic agents are critical. Aurora kinases play a crucial role in mitosis and selective inhibitors might provide an effective therapeutic treatment for cancer. However, the role of Aurora kinases in EC is still inadequately studied. Here, we identified a novel compound, referred to as APIO-EE-9, which inhibits growth and colony formation and induces apoptosis of esophageal cancer cells. Using computer modeling, we found that APIO-EE-9 interacted with both Aurora A and B in the ATP-binding pocket. APIO-EE-9 inhibited both Aurora A and B kinase activities in a dose-dependent manner. Treatment with APIO-EE-9 substantially reduced the downstream Aurora kinase phosphorylation of histone H3 (Ser10), resulting in formation of multiple nuclei and centrosomes. Additionally, esophageal cancer cells expressing shAurora A or shAurora B kinase exhibited a dramatic reduction in proliferation and colony formation. Injection of these cells as xenografts in mice reduced tumor formation compared to wildtype cells. Importantly, APIO-EE-9 significantly decreased the size of esophageal patient-derived xenograft (PDX) tumors implanted in SCID mice. These results demonstrated that APIO-EE-9 is a specific Aurora kinase inhibitor that could be developed as a therapeutic agent against esophageal cancer.

## INTRODUCTION

Esophageal cancer (EC) is one of the most lethal cancers worldwide with its incidence on the rise. Each year, more than 450,000 new cases are diagnosed [1]. In China, EC mortality and morbidity rates are 4^th^ and 6^th^ highest, respectively [2]. According to the Surveillance, Epidemiology, and End Results Program database, in the United States in the past few decades, the increased incidence of EC malignancy is greater than that seen for melanoma, breast cancer, or prostate cancer. The increase in incidence was observed for all stages [3, 4]. Therefore, EC represents an important global health issue. Despite the use of modern surgical techniques in combination with radiotherapy and chemotherapy, the overall 5-year survival rate remains below 20% [5, 6]. The recent development of targeted therapies could provide promise for improved esophageal cancer treatment and management.

The Aurora serine/threonine kinases regulate cell cycle and have been reported to be overexpressed in many types of tumors, including multiple myeloma, colorectal, prostate, and pancreatic cancers [7, 8]. The overexpression has made the Aurora kinases attractive targets for developing therapeutic cancer drugs [9].

In mammals, the Aurora kinase family comprises 3 members, Aurora A, B, and C [10]. However, they display distinct functional differences during mitosis, which is reflected in their subcellular locations and activities. Aurora A has been characterized as a mitotic kinase and is required for centrosome maturation, separation and spindle assembly [8, 11] and is localized at the spindle poles [12]. Small molecule inhibitors of Aurora A cause defects in centrosome separation with the formation of multiple centrosomes [13]. Aurora B is a chromosomal passenger protein that can move from centromeres to the spindle mid-zone during mitosis [14]. Aurora B localizes to the chromosome in prophase, the centromere in pro-metaphase and metaphase, and to the central mitotic spindle in anaphase [15–17]. Aurora B is required for phosphorylation of histone H3 [18], chromosome condensation, kinetochore functions, spindle checkpoint activation and cytokinesis completion [19]. Suppressing Aurora kinase activity with small molecule inhibitors causes failure in cytokinesis and abnormal exit from mitosis, resulting in polyploid cells and ultimately apoptosis [13, 20]. Less is known about Aurora C compared with the other Aurora kinases. However, Aurora C is known to be highly expressed in the testes and to play a key role in meiosis and, more specifically, in the regulation of spermatogenesis [17, 21].

Many cancer types display a lack of cell cycle and cell cycle checkpoint regulation, resulting in diseases characterized by uncontrolled proliferation [11]. Both Aurora A and B are associated with cell cycle checkpoint regulation and mitosis, and therefore, have been implicated in oncogenesis [22]. Currently, several Aurora kinase inhibitors have entered into clinical trials and include AZD1152 [23], MLN8237 [24, 25], VX-680 [26]. However, a number of these inhibitors have show toxicity or other undesirable side effects. Thus, identifying small-molecule inhibitors of Aurora kinases with fewer side effects is crucial for testing in translational studies and early-phase clinical trials to facilitate the development of potential drugs for cancer therapy.

Herein, we report that a newly synthesized Aurora kinase inhibitor, referred to as APIO-EE-9, suppresses esophageal cancer cell growth by inhibiting Aurora kinase A or B activity and downstream phosphorylation of histone H3 *in vitro* and in cells. Based on this information, we knocked down Aurora A or Aurora B expression to study the function of Aurora A and B kinases in esophageal cancer. Results showed that esophageal cancer cell growth was inhibited after knocking down Aurora A or B expression. Moreover, APIO-EE-9 strongly inhibited esophageal tumor growth in a patient-derived xenograft (PDX) mouse model. Overall, our data showed that APIO-EE-9 exerts antitumor activities by targeting Aurora A and B, providing a potential therapy against esophageal cancer.

## RESULTS

### APIO-EE-9 inhibits anchorage-independent growth and proliferation of esophageal cancer cells

Aurora A is reportedly overexpressed in several types of human tumors, including ovarian cancer [27], breast cancer [28], and gastric cancer [29]. Abnormal Aurora A expression also contributes to esophageal cancer development and cisplatin resistance [30, 31]. The scientific literature indicates that high levels of Aurora A kinase are associated with advanced clinical stage and poor prognosis in several cancers [32–36]. Additionally, Aurora B overexpression is associated with acute myeloid leukemia [37] and colorectal cancer [38].

**Figure 1:**
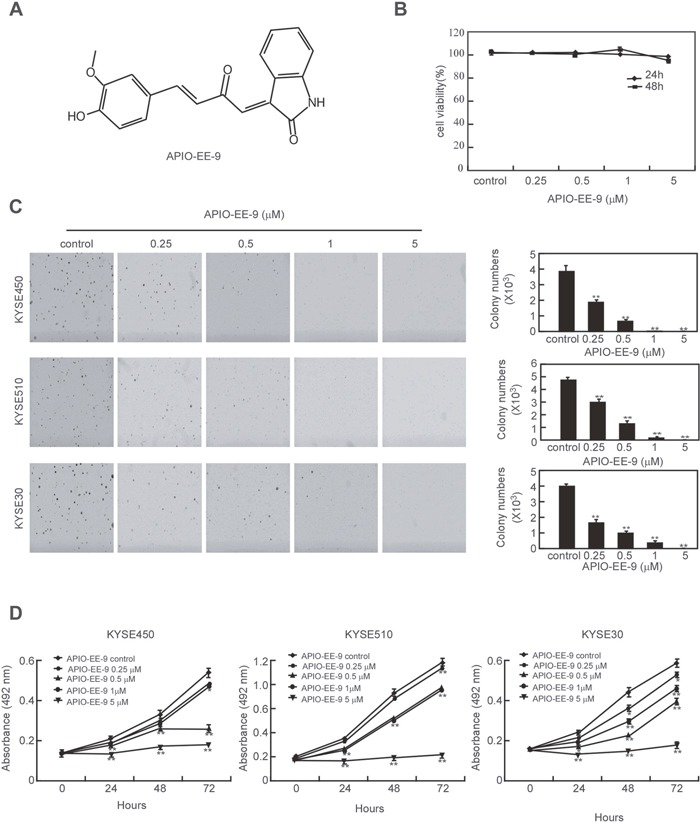
APIO-EE-09 inhibits anchorage-independent growth and viability of esophageal cancer cell **(A)** Chemical structure of APIO-EE-9. **(B)** Cytotoxicity of APIO-EE-9 was assessed by MTS assay in the normal Het-1A esophageal cancer cell line. **(C)** KYSE450, KYSE30 and KYSE510 esophageal cancer cell lines were treated or not treated with various concentrations of APIO-EE-9 and anchorage-independent growth was measured. Data are shown as mean values ± S.D. from triplicate experiments. The asterisks (**) indicate a significant (*p* < 0.01) decrease in colony formation compared to the control group. **(D)** KYSE450, KYSE30 and KYSE510 esophageal cancer cells were treated with different concentrations of APIO-EE-9 and viability of estimated. Data are shown as means ± S.D. from triplicate experiments. The asterisks (*, **) indicate a significant (*p* < 0.05, *p <* 0.01, respectively) decrease in viability compared to the control group.

By screening a large in-house compound database, APIO-EE-9 was identified (Figure 1A, Table 1) as a novel antagonist against either Aurora A or B and was selected for further study as a potential therapeutic drug against esophageal cancer. Normal Het-1A esophageal cells and esophageal cancer cells were used to determine the cytotoxicity of APIO-EE-9. Results showed that APIO-EE-9 exhibits no cytotoxicity against normal Het-1A cells (Figure 1B) whereas cancer cells were sensitive to APIO-EE-9 treatment (Supplementary Figure 1A). Next, the KYSE450, KYSE510 and KYSE30 esophageal cancer cell lines and normal Het-1A esophageal cells were treated with different concentrations of APIO-EE-9 and colony formation was assessed. Results demonstrated that APIO-EE-9 dramatically inhibited colony formation (Figure 1C) and decreased viability (Figure 1D) in a dose-dependent manner, but it had little effect on the proliferation of normal cells (Supplementary Figure 1B).

**Table 1:** Kinase docking results indicate that Aurora A and B are potential targets of APIO-EE-9

Kinase	Docking	Kinase	Docking	Kinase	Docking
	Score		Score		Score
**Aurora B**	**-9.212**	RSK1	−7.73	PIM1	−6.813
PDK1	−9.058	EGFR	−7.684	AKT2	−6.772
ERK1	−8.797	MEK2	−7.682	EGFR (L858R)	−6.675
MLK1	−8.793	c-MET	−7.457	RSK2 (CTD)	−6.596
**Aurora A**	**-8.597**	CHK2	−7.433	RSK2 (NTD)	−6.15
PI3 Kinase (p110/p85)	−8.424	ALK	−7.154	GSK3β	−5.753
EGFR (T790M)	−8.404	JNK1	−7.15	AKT1	−5.102
MSK1	−8.19	P38	−7.095	ERK2	−5.01
ASK1	−7.911	MEK1	−6.909	CHK1	−4.371

### APIO-EE-9 induces apoptosis of esophageal cancer cells

**Figure 2:**
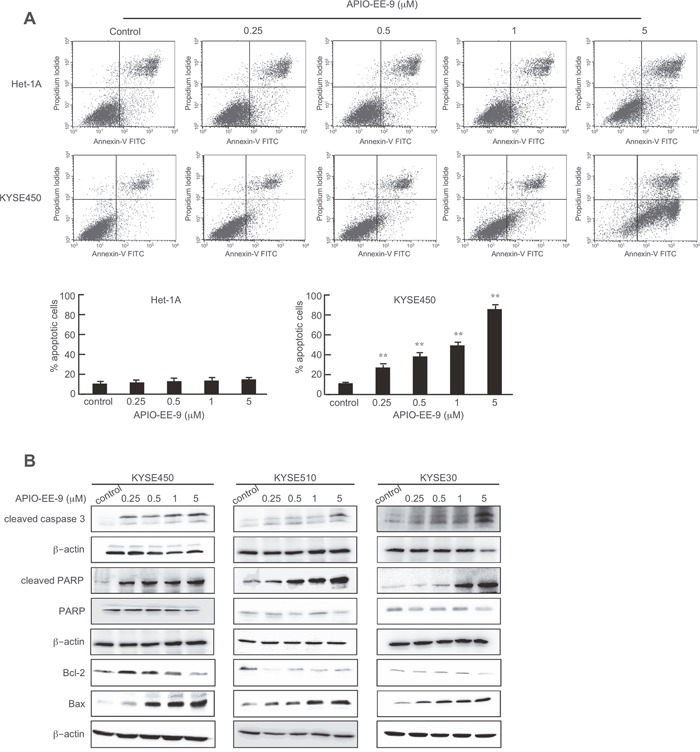
APIO-EE-9 induces apoptosis of esophageal cancer cell lines **(A)** Normal esophageal Het-1A cells or KYSE450 esophageal cancer cells were treated with different concentrations of APIO-E-9 for 72 h and then stained with annexin V. Apoptosis was analyzed by flow cytometry. The asterisks (**) indicate a significant (*p* < 0.01) increase in percentage of cells undergoing apoptosis. **(B)** KYSE450, KYSE510, or KYSE30 esophageal cancer cells were treated with different concentrations of APIO-EE-9 for 72 h. Cells were harvested and cleaved caspase 3, Bcl-2, Bax and cleaved PARP proteins were detected by Western blotting using specific antibodies. β-Actin was used as a loading control and each experiment was repeated at least 3 times with similar results.

We assessed the effect of APIO-EE-9 treatment on apoptosis of Het-1A and KYSE450 cells. Data illustrated that APIO-EE-9 caused significant apoptosis in the KYSE450 cell line (Figure 2A, middle and lower panels), but has no effect on normal Het-1A esophageal cells (Figure 2A, upper and lower panels). Furthermore, KYSE450, KYSE510, and KYSE30 esophageal cancer cells treated with the compound exhibited substantial up-regulation of apoptosis-associated proteins, including cleaved-PARP, cleaved caspase 3 and pro-apoptotic Bax whereas anti-apoptotic Bcl-2 expression was decreased (Figure 2B).

### Aurora A and B are highly expressed in esophageal cancer cell lines and tissues

**Figure 3:**
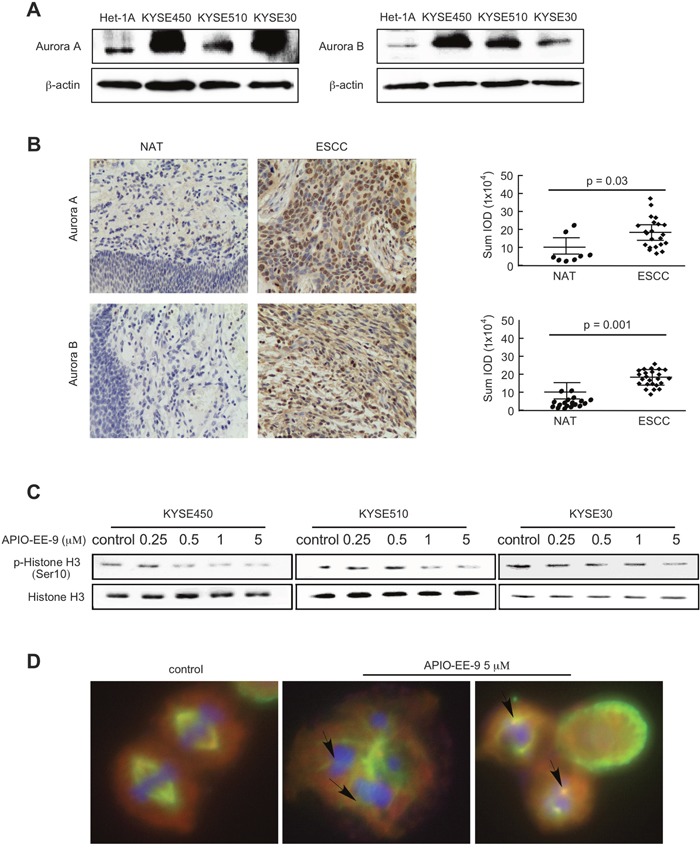
Esophageal cancer cells and tissues highly express Aurora A or Aurora B kinase **(A)** KYSE450, KYSE510 or KYSE30 esophageal cancer cells and normal Het-1A esophageal cells were harvested and Aurora A and B expression was detected by Western blot. **(B)** Immunohistochemical (IHC) staining of Aurora A and B in esophageal tumor tissues. The integrated optical density (IOD) was evaluated using the Image-Pro Plus software (v. 6.2) program. The asterisks (*, **) indicate a significant difference (*p <* 0.05, *p* < 0.01) in tumor staining of Aurora A or B compared with the adjacent normal esophageal tissue (NAT). **(C)** KYSE450, KYSE510, or KYSE30 esophageal cancer cells were treated with different concentrations of APIO-EE-9 for 24 h. Cells were harvested and phosphorylation of histone H3, a downstream substrate of Aurora B, was detected by Western blotting. **(D)** APIO-EE-09 induced polyploidy and multiple centrosome formation in esophageal cancer cell lines. KYSE450 cells were stained with DAPI, γ-tubulin, or α-tubulin, after treatment with 5 μM APIO-EE-9 for 2 h (nucleus = blue; centrosome = green; cytoskeleton = red).

Aurora kinases are highly overexpressed in many types of cancers [39]. Expression of Aurora A and B was compared in normal Het-1A esophageal cells and KYSE450, KYSE510 and KYSE30 esophageal cancer cells. Western blot (WB) results showed that both Aurora A and B are highly expressed in esophageal cancer cell lines compared with normal cells (Figure 3A). IHC staining of esophageal tissues was used to examine Aurora A or B expression in patient samples. Results reveal that Aurora A or B is highly expressed in esophageal cancer tissues compared with normal adjacent tissues (NAT; Figure 3B, Table 2). Histone H3 (Ser10) has been reported to be phosphorylated by Aurora kinases and is required for proper chromosome dynamics during mitosis [8]. To better understand the relationship between APIO-EE-9 and the Aurora kinases, we used Western blot analysis to determine the level of phosphorylated histone H3 (Ser10) after treatment with various concentrations of APIO-EE-9. Results demonstrated that APIO-EE-9 inhibits histone H3 (Ser10) phosphorylation in a dose-dependent manner (Figure 3C). This suggests that APIO-EE-9 might target the Aurora kinases. Aurora A reportedly regulates centrosome maturation and separation [40], whereas Aurora B kinase is a chromosomal passenger protein critical for accurate chromosomal segregation and cytokinesis [20]. Therefore, if Aurora kinases are a target of APIO-EE-9, treatment with this compound should induce multi-nucleation and multi-centrosome formation. Immunofluorescence results revealed that APIO-EE-9 does indeed induce multi-nucleation and multi-centrosome formation in KYSE450 cells (Figure 3D, Supplementary Figure 1C). Overall, these data illustrate that Aurora A or B kinase might be a potential target for APIO-EE-9.

**Table 2:** Expression of Aurora A and Aurora B in ESCC tissues

Parameter	n	Aurora A expressionIOD sum (X10^4^)	*P* value
Histological grade			0.03*
II	8	20 ± 7	
III	14	15 ± 2	
TNM stage			0.03*
II	4	23 ± 7	
III	18	15 ± 1	
Histological grade			0.001**
II	9	15 ± 7	
III	15	21 ± 2	
TNM stage			0.001**
II	4	16 ± 6	
III	20	19 ± 2	

*, ** indicates statistical significance (*p* ≤ 0.05, *p* ≤ 0.01).

### APIO-EE-9 inhibits Aurora A and B kinase activities *in vitro* and *ex vivo*

**Figure 4:**
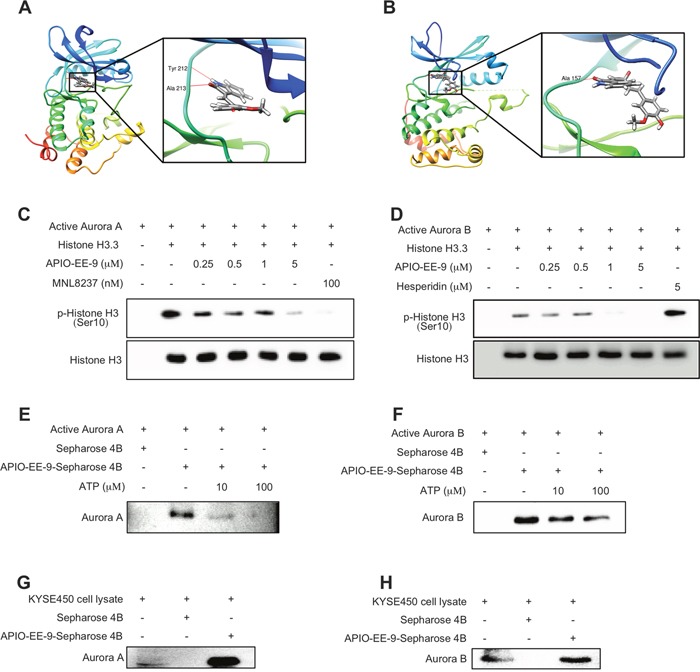
APIO-EE-9 inhibits both Aurora A and B kinase activities *in vitro* and *ex vivo* **(A)** Modeling of APIO-EE-9 binding with Aurora A. APIO-EE-9 binding with Aurora A at the ATP binding pocket (*left*) and an enlarged view of the binding (*right*). **(B)** Modeling of APIO-EE-9 binding with Aurora B. APIO-EE-9 binding with Aurora B at the ATP binding pocket (*left*) and an enlarged view of the binding (*right*). APIO-EE-9 inhibits Aurora A **(C)** and B **(D)** kinase activities *in vitro*. Active kinase Aurora A or B and different doses of APIO-EE-9 were incubated at 37°C for 5 min with histone H3.3 as substrate. Phosphorylation of histone H3.3 (Ser10) was detected by Western blot. The *in vitro* **(E, F)** and *ex vivo* ATP competitive binding **(G, H)** of APIO-EE-9 with Aurora A or B were confirmed by pull-down assay. Active Aurora A or B (200 ng) or a KYSE450 cell lysate (500 μg) and various concentrations of ATP were incubated with APIO-EE-9-Sepharose 4B beads (or Sepharose 4B beads only as a control). Aurora kinase A or B protein expression was detected by Western blotting.

To better understand how APIO-EE-9 interacts with Aurora A or B, we docked the compound into the respective ATP binding pocket of Aurora A or B, using protocols in the Schrödinger Suite 2015. Results of the final computational model docking result showed that APIO-EE-9 formed hydrogen bonds with Aurora A and B at their respective ATP binding pocket (Figure 4A, 4B, respectively; images were generated with the UCSF Chimera program [41]. This indicates that APIO-EE-9 might be a potential inhibitor of Aurora A or B. To further examine the mechanism of APIO-EE-9's inhibitory effect, we used *in vitro* kinase assays to determine the effect of APIO-EE-9 on the Aurora kinases. Active Aurora A (100 ng) or B (50 ng) was mixed with histone H3.3. Then different amounts of AOPIO-EE-9, MNL8237 or Hesperidin (positive control) were added and the mixtures incubated at 30°C for 30 or 5 min, respectively. Results showed that 5 μM APIO-EE-9 completely inhibited the activity of Aurora A or B (Figure 4C, 4D). To further verify this result, we conjugated APIO-EE-9 with Sepharose 4B beads and conducted a pull-down assay. We confirmed that active Aurora A or B kinase binds with APIO-EE-9-Sepharose 4B beads, but not with Sepharose 4B beads alone (Figure 4E, 4F). Results of an ATP competition assay showed that the binding ability of APIO-EE-9 with Aurora A or B (Figure 4E, 4F) was decreased in the presence of ATP, indicating competition. Using KYSE450 cell lysates, we performed an *ex vivo* pull-down assay and results revealed that APIO-EE-9 also binds with Aurora A or B in esophageal cancer cells (Figure 4G, 4H). Overall, our results clearly demonstrated that APIO-EE-9 inhibits Aurora A or B kinase activity in a dose-dependent manner competitive with ATP.

### Knocking down Aurora A or B expression strongly inhibits esophageal cancer cell anchorage-independent growth and proliferation and induces apoptosis

**Figure 5:**
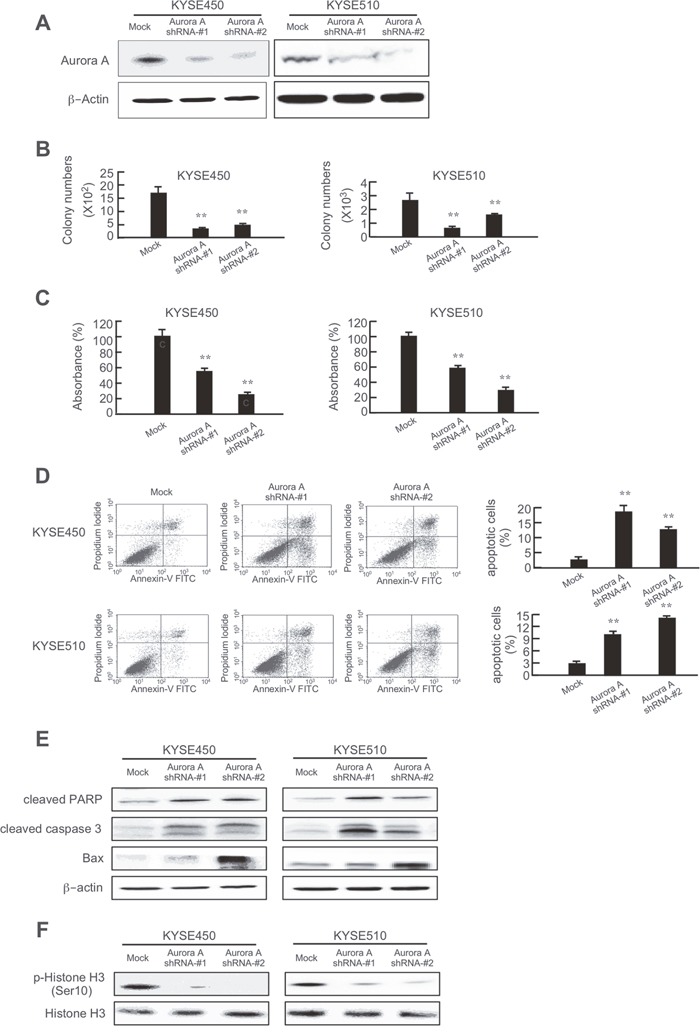
Knocking down Aurora A expression in esophageal cancer cells inhibits anchorage-independent growth and proliferation and induces apoptosis **(A)** Efficiency of Aurora A shRNA in KYSE450 and KYSE510 cells. Knocking down Aurora A expression in KYSE450 and KYSE510 cells inhibits **(B)** colony formation and **(C)** proliferation and increases **(D)** apoptosis. **(E)** Knocking down Aurora A increases pro-apoptotic protein expression and decreases anti-apoptotic protein expression. **(F)** Knocking down Aurora A inhibits phosphorylation of histone H3 (Ser10). The asterisks (**) indicate a significant (*p* < 0.01) difference in shRNA-transfected cells compared with the mock-transfected group.

**Figure 6:**
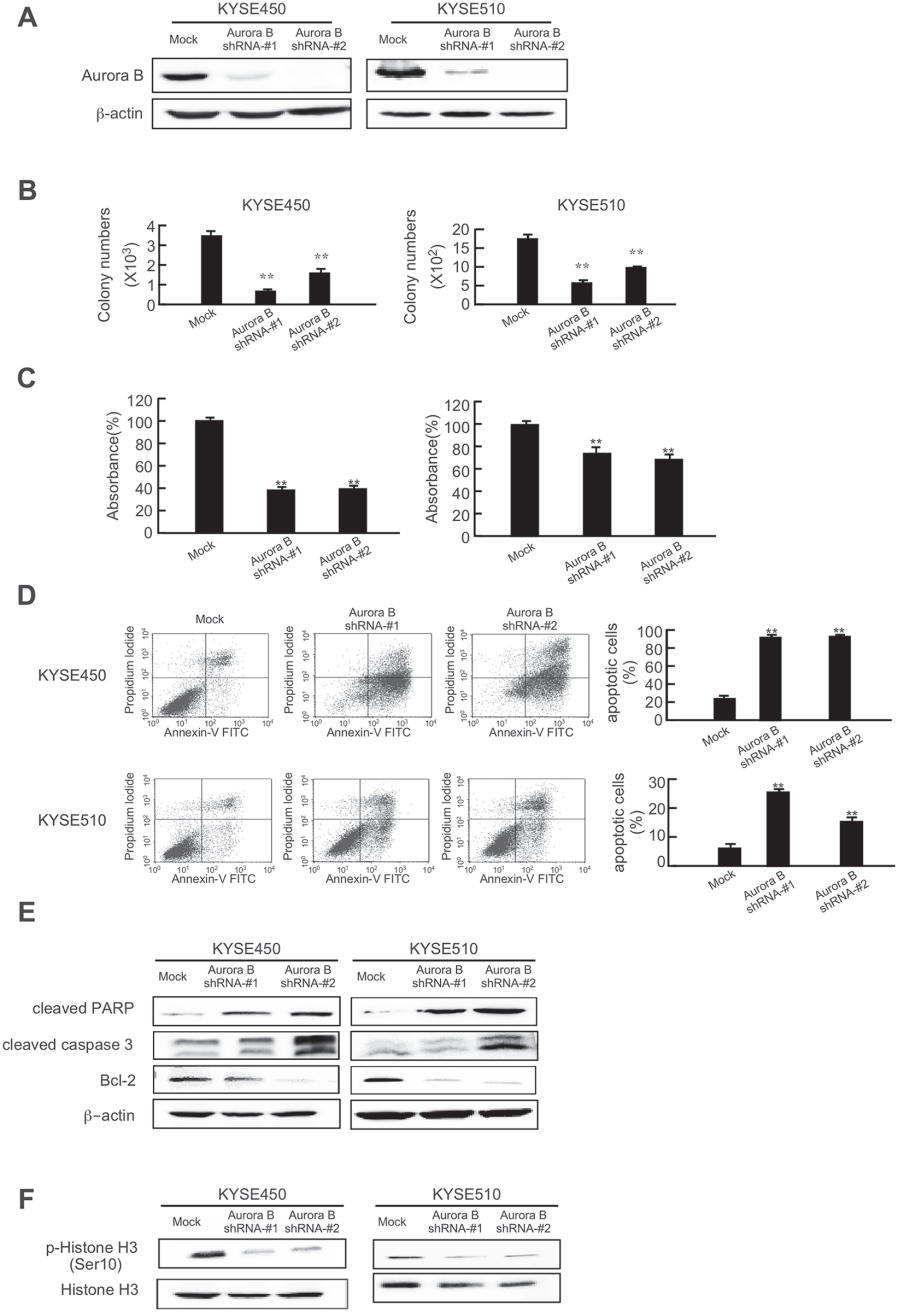
Knocking down Aurora B expression in esophageal cancer cells inhibits anchorage-independent growth and proliferation and induces apoptosis **(A)** Efficiency of Aurora B shRNA in KYSE450 and KYSE510 cells. Knocking down Aurora B expression in KYSE450 and KYSE510 cells inhibits **(B)** colony formation and **(C)** proliferation and increases **(D)** apoptosis. **(E)** Knocking down Aurora B increases pro-apoptotic protein expression and decreases anti-apoptotic protein expression. **(F)** Knocking down Aurora B inhibits phosphorylation of histone H3 (Ser10). The asterisks (**) indicate a significant (*p* < 0.01) difference in shRNA-transfected cells compared with the mock-transfected group.

Because Aurora A and B mitotic kinases drive cell division and are overexpressed in tumors with a high mitotic index, we hypothesized that knocking down Aurora A or B expression would produce an antitumor effect against esophageal cancer cells. First, the efficiency of short hairpin RNA (shRNA) knockdown in KYSE450 and KYSE510 esophageal cancer cells was examined and results showed that the expression of Aurora A or B was obviously decreased after shRNA transfection (Figures 5A, 6A). Moreover, colony formation (Figures 5B, 6B, Supplementary Figure 1D) and cell growth (Figures 5C, 6C) were dramatically inhibited in the shRNA-Aurora A- (Figure 5B, 5C) or shRNA-Aurora B- (Figure 6B, 6C) transfected groups compared with the negative control (mock-transfected groups). Inducing apoptosis is recognized as a crucial strategy for effective cancer therapy. Apoptosis was increased in KYSE450 and KYSE510 esophageal cancer cells after Aurora A (Figure 5D) or B (Figure 6D) protein expression was attenuated by shRNA transfection. Knocking down Aurora A or B expression with shRNA resulted in increased expression of pro-apoptotic markers, including cleaved caspase 3, cleaved PARP and Bax and decreased expression of anti-apoptotic Bcl-2 (Figures 5E, 6E). In addition, knocking down Aurora A or B also inhibited phosphorylation of histone H3 (Ser10) in esophageal cancer cells (Figures 5F, 6F). Overall, these data showed that Aurora A and B play a critical role in KYSE450 and KYSE510 esophageal cancer cell growth.

### Knocking down Aurora A and Aurora B expression dramatically suppresses tumor growth in an *in vivo* mouse model

**Figure 7:**
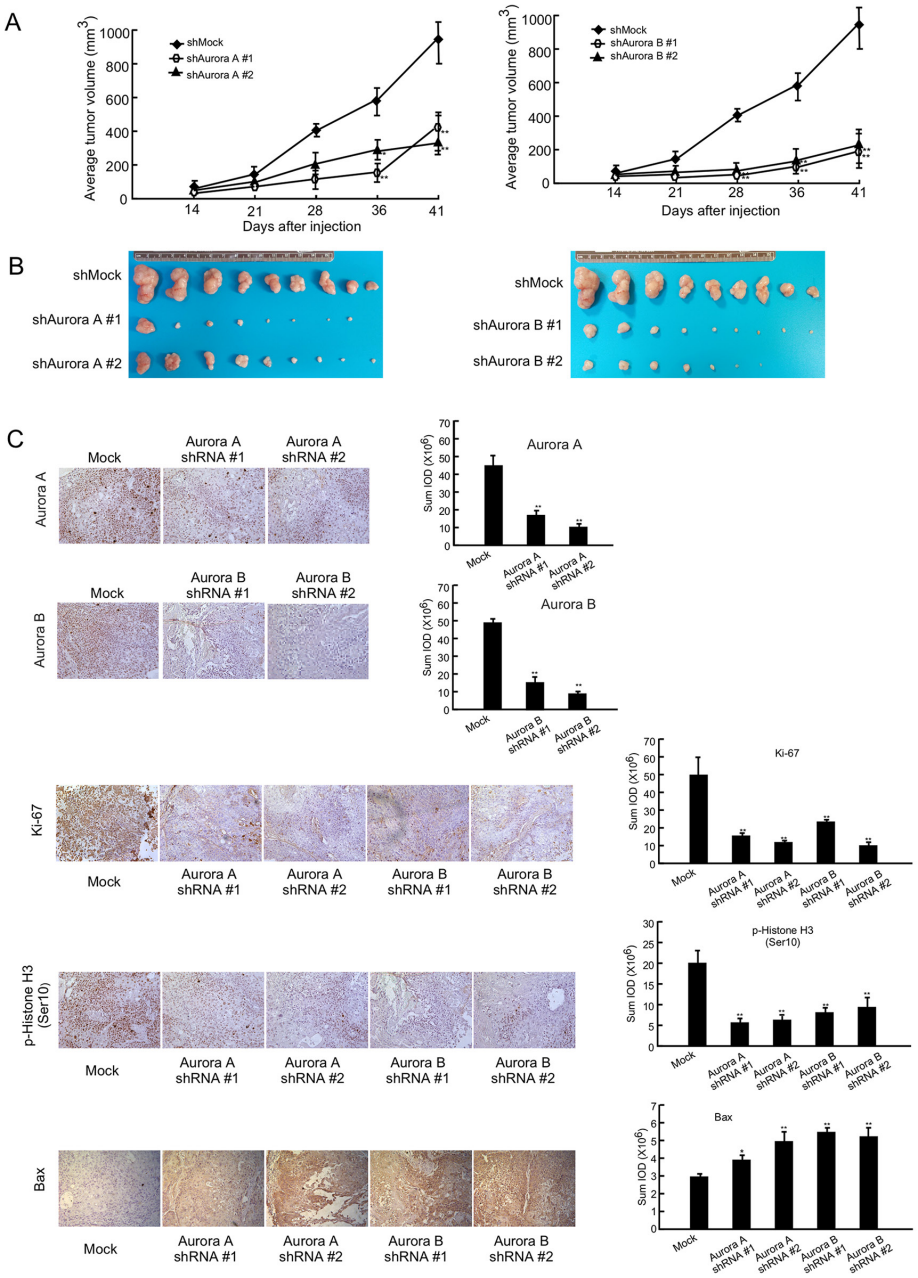
Knocking down Aurora A or B expression in KYSE450 esophageal cancer cells dramatically inhibits tumor growth in a xenograft mouse model Mice injected with knockdown cells exhibited decreased tumor volume **(A)** and weight **(B)** compared with mice injected with mock. The asterisks (*, **) indicate a significant (*p* < 0.05, *p* < 0.01, respectively) decrease in tumor volume. **(C)** Immunohistochemistry analysis was used to determine the level of Ki-67, phosphorylation of histone H3 (Ser10) and Bax. The integrated optical density (IOD) was evaluated using the Image-Pro Plus (v.6.2). The asterisks (*, **) indicate a significant (*p* < 0.05, *p* < 0.01, respectively) decrease in the shRNA transfection groups compared with mock control.

To further study the function of Aurora A and B, we performed an *in vivo* xenograft experiment. Athymic nude mice were injected with KYSE450 cells expressing knock down Aurora A or B. Results showed that knocking down Aurora A or Aurora B expression suppressed KYSE450-xenograft tumor growth (Figure 7A). After mice were sacrificed, tumor size was measured and results showed that tumor size was dramatically inhibited in mice implanted with shRNA expressing cells (Figure 7B, Supplementary Figure 1E). To confirm that the antitumor effect of APIO-EE-9 was associated with inhibition of Aurora A or B, an immunohistochemical analysis was performed. Data showed that Aurora A or B expression level was obviously decreased after shRNA transfection. Ki-67 and phosphorylation of histone H3 were dramatically inhibited and Bax protein expression was significantly increased in the shRNA groups compared with the Mock group (Figure 7C). Overall, these data clearly indicated that APIO-EE-9 anticancer effects are mainly associated with the suppression of Aurora A or B activation.

### APIO-EE-9 suppresses esophageal tumor growth in a PDX mouse model

**Figure 8:**
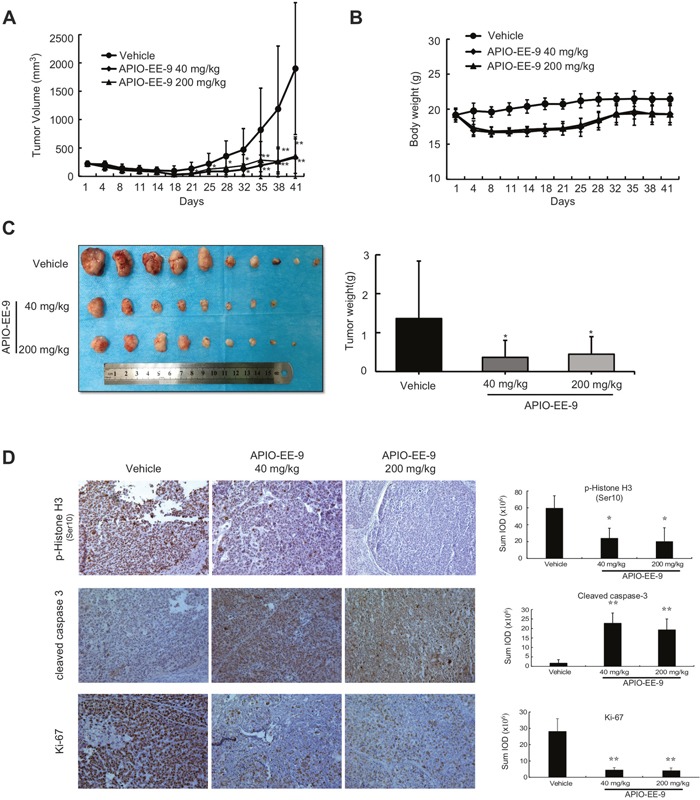
APIO-EE-9 suppresses esophageal PDX tumor growth **(A)** Treatment with APIO-EE-9 inhibits ESCC PDX tumor size compared with the untreated group. The asterisks (*, **) indicate a significant (*p* < 0.05, *p* < 0.01, respectively) decrease in tumor volume in the APIO-EE-9-treated groups compared with the vehicle-treated group. **(B)** Mouse tumors were photographed (*upper*) and tumor weight determined (*lower*). The asterisk (*) indicates a significant (*p* < 0.05) decrease in tumors from mice treated with APIO-EE-9 compared with the vehicle-treated group. **(C)** Mouse body weight. **(D)** Immunohistochemistry (IHC) staining of phosphorylated (p)-histone H3 (Ser10), cleaved caspase 3, and Ki-67. The relative integrated optical density (IOD) value was obtained using the Image-Pro Plus software program. The asterisks (*, **) indicate a significant (*p* < 0.05, *p* < 0.01, respectively) decrease in staining in tissues from mice treated with APIO-EE-9 compared with vehicle-treated mice.

In addition, we conducted patient-derived xenograft (PDX) studies with tumor tissues collected from esophageal cancer patients with high expression of Aurora A and B to further investigate the effectiveness of APIO-EE-9 (Supplementary Figure 2A). The results showed that APIO-EE-9 (40 or 200 mg/kg) effectively inhibited PDX tumor growth compared with the vehicle-treated group (Figure 8A, 8B) with no significant loss in body weight (Figure 8C), suggesting minimal toxicity. In addition, we conducted another PDX study with tumor fragments also collected from esophageal cancer patients. The results showed that injection of APIO-EE-9 (40 mg/kg) in mice significantly suppressed the growth of esophageal cancer tumors (Supplementary Figure 2B, 2C). Again, no significant changes were observed in mouse body weight (Supplementary Figure 2D). These data agreed with the previously obtained results described above (Figures 7, 8). Additionally, treatment with APIO-EE-9 suppressed Ki-67 and phosphorylation of histone H3 expression and increased cleaved caspase 3 levels (Figure 8D). Overall, these results illustrated that APIO-EE-9 has potential as chemotherapeutic agent against esophageal cancer.

## DISCUSSION

The two main types of esophageal cancer (EC) comprise esophageal squamous cell carcinoma (ESCC) and esophageal adenocarcinoma (EA) [42]. ESCC is the primary type of esophageal cancer and is characterized by a poor prognosis and high invasiveness and extends geographically from North-Central China to the Middle East [43]. An increased risk of ESCC is associated with tobacco smoking, excessive alcohol consumption, drinking mate (i.e., a traditional South American caffeine-rich infused drink), low intake of fresh fruits and vegetables, achalasia, and low socioeconomic status. Most patients with esophageal cancer are diagnosed at advanced (metastatic) stages and thus have a poor prognosis [5]. Because of its aggressive nature, esophageal cancer remains a challenging disease to treat. Despite advances in surgical techniques and multi-modality therapies, the 5-year survival rate remains poor (14%). Tumor markers of esophageal cancer are an advancing area of research that could potentially lead to earlier diagnosis as well as play a part in assessing tumor response to therapy. Various reports have identified cancer relevant pathways, including the vascular endothelial growth factor (VEGF) receptor [44, 45], the cyclooxygenase-2 (COX-2) [46, 47], epidermal growth factor receptor (EGFR) [48], and mammalian target of rapamycin (mTOR) [49], as EC treatment targets. However, targeting these pathways had limited promising effects on survival rate in patients. Targeted therapy is a type of chemotherapy that takes advantage of genetic differences between normal and cancer cells. Our results revealed that compared with normal samples, both Aurora A and B are highly expressed in esophageal cancer cells and tissue samples (Figure 3A, 3B), suggesting that the Aurora kinases might be promising molecular markers for EC treatment. Aurora kinases have been reported to play a key role in mitosis [50]. According to the literature, Aurora A is a cell cycle-regulated kinase in spindle formation [34] and Aurora B is a chromosomal passenger protein critical for accurate chromosomal segregation, cytokinesis and regulation of the mitotic checkpoint [20]. Deregulation of Aurora kinase activity can lead to mitotic abnormality and genetic instability, leading to defects in centrosome function, spindle assembly, chromosome condensation, microtubule-kinetochore attachment, and cytokinesis [51–53], which can contribute to oncogenesis. Aurora A overexpression is associated with aneuploidy, supernumerary centrosomes, defective mitotic spindles, and resistance to apoptosis [54]. Aurora B deregulation leads to progression through anaphase despite the presence of misaligned chromosomes [35]. In addition, amplification of Aurora A was detected in 27 of 29 (93.1%) esophageal cancer samples [30, 54]. These data reveal that gene amplification might be a major cause of Aurora A overexpression in esophageal cancer. However, some reports demonstrated that discordance exists between gene amplification and expression of Aurora B in malignant mesothelioma [55], glioblastoma [56], colon cancer, prostatic cancer and pancreatic cancer [57]. This suggests that other mechanisms are involved in the increased Aurora B gene expression, such as transcriptional activation in esophageal cancer. Overall, Aurora kinases have emerged as attractive therapeutic targets for esophageal cancer treatment.

Over the past decade, the field of drug development has been transformed with the identification and capability to direct treatments at specific molecular targets. However, the number of approved targeted agents remains few and novel agents have not yet been widely explored in esophageal cancer. In this study, we identified an important and novel small molecule inhibitor referred to as APIO-EE-9, which attenuated both Aurora A and B kinase activities (Figure 4C, 4D). Multinuclear and multiple centrosome formation appeared after treatment with a high dose (5 μM) of APIO-EE-9 (Figure 3D). A series of Aurora kinase inhibitors have been discovered and developed with the intent of inhibiting Aurora A and B [9, 58, 59]. For example, Hesperidin acts solely as a relatively selective Aurora B inhibitor with a low affinity to Aurora A. Meanwhile, another compound, MLN8237, was discovered to perform an opposite function. Because each has low cytotoxicity and specific therapeutic targets, some have entered clinical trials. For example, MLN8237 is presently undergoing Phase I and Phase II clinical trials administered solely or in combination with other agents as a cancer treatment [60, 61]. On the other hand, results of Phase I studies of AZD1152 revealed that it had dose-limiting toxicity to neutropenia patients [62]. In addition, in some clinical trials a limited efficacy against solid tumors was observed based on the wide use of different chemical classes of Aurora kinase inhibitors [62]. Therefore, finding Aurora kinase inhibitors that can suppress both Aurora A and B activity with fewer side effects are needed and additional mechanistic and clinical studies are warranted. Our data showed that APIO-EE-9 significantly decreased esophageal cancer cell growth (Figure 1B, 1D) and induced apoptosis (Figure 2A), but did not have any effect on normal esophageal Het-1A cells. Importantly, an *in vivo* PDX animal model showed that APIO-EE-9 (40 or 200 mg/kg) effectively inhibited tumor growth compared with the vehicle-treated group with no significant toxicity (Figure 8A-8C). Meanwhile, none of the mice showed any obvious signs of adverse side effects (Figure 8B), which suggests that this compound was well tolerated. These data were well aligned with previous Aurora kinase inhibitor reports. No significant toxicity or adverse side effects were observed [63], and importantly, most of the side effects were reversible upon drug withdrawal [64]. However, additional larger scale studies will be needed in order to evaluate any potential for undesirable side effect in order to move APIO-EE-9 forward for clinical development.

Overall, we showed that both Aurora A and B are highly expressed in esophageal cancer cell lines. A newly identified small molecule, referred to as APIO-EE-9, potently and dose-dependently inhibits Aurora A and B kinase activities. This compound is an effective and novel Aurora A and B inhibitor that could provide a new agent for esophageal cancer therapy and needs further investigation and development.

## MATERIALS AND METHODS

### Chemicals and reagents

Compound APIO-EE-9 was synthesized per the protocol reported for similar compounds but with some modifications [65]. CNBr-activated Sepharose^TM^ 4B beads were purchased from GE Healthcare Bio-Sciences (Uppsala, Sweden). The antibodies against β-actin and α-tubulin were from Santa Cruz Biotechnology (Santa Cruz, CA). The Aurora A and B antibodies were obtained from Abcam (Cambridge, MA). The antibodies to detect total and phosphorylated histone H3 (Ser10) were from Cell Signaling Biotechnology (Beverly, MA) and all active kinases were from EMD Millipore Corporation (Billerica, MA).

### Cell culture

KYSE450, KYSE30 and KYSE510 human esophageal cancer cell lines, normal Het-1A esophageal cells, and 293T cells were purchased from American Type Culture Collection (ATCC; Manassas, VA). All cells were cytogenetically tested and authenticated before freezing and were cultured at 37°C in a 5% CO_2_ incubator according to ATCC protocols. The KYSE450, KYSE30, KYSE510 cell lines were cultured in RPMI 1640 medium/10% FBS and the 293T cell line was cultured in DMEM/10% FBS. The normal Het-1A cell line was cultured in L-15 medium with 5 μg/ml collagen type I and 30 mg/ml BSA.

### MTS assay

Cells (1 × 10^3^ or 5 × 10^3^ per well) were seeded in 96-well plates in a final volume of 100 μl each well to determine proliferation or cytotoxicity, respectively. After 24 h incubation, cells were treated with different concentrations of APIO-EE-9 and harvested at various times. The CellTiter96 Aqueous One Solution (20 μl; Promega Corporation, Madison, WI) was added to each well and cells were incubated for an additional 1 h at 37°C. The absorbance was then measured at 492 nm by spectrophotometer.

### Anchorage-independent cell growth assay

Cells (8 × 10^3^) were suspended in 1 ml BME/10% FBS/0.33% agar with different concentrations of APIO-EE-9 and plated on 3 ml solidified BME/10% FBS/0.5% agar with the same concentration of APIO-EE-9 in each well of 6-well plates. The cultures were maintained at 37°C in a 5% CO_2_ incubator for 1–2 weeks and then colony numbers were counted under a microscope using the Image-Pro Plus software program (Media Cybernetics, Inc. Rockville, MD).

### Apoptosis assay

Cells (1.5 × 10^5^) were plated in 60-mm dishes and incubated overnight and then treated for 72 h with different concentrations of APIO-EE-9. The cells were fixed with ice-cold 70% ethanol at −20°C overnight. After staining with Annexin V, apoptosis was analyzed by 2-color flow cytometry.

### Western blot analysis

The protein concentration of samples was measured using a protein assay kit (Bio-Rad Laboratories, Hercules, CA). Proteins were resolved by SDS-PAGE and transferred onto polyvinylidene difluoride membranes (Millipore), which were blocked with nonfat milk and hybridized with specific primary antibodies at 4°C overnight. The protein bands were visualized using the enhanced chemiluminescence reagent after hybridization with a horseradish peroxidase–conjugated secondary antibody.

### Immunofluorescence

Cells (2×10^4^) were seeded into 4-well chamber slides (Millipore, cat. # PEZGS0416) and incubated overnight. They were then treated with a high dose (5 μM) of APIO-EE-9 for 2 h, then fixed with cold methanol at −20°C for 20 min followed by soaking in 1% PBS/BSA with 0.3% Tritonx-100 for 30 min at room temperature. Antibodies were diluted in 1% PBS-BSA. A mouse anti-human α-tubulin (1:400, Santa Cruz Biotechnology) antibody and 488-conjugated goat anti-mouse IgG secondary antibody were used to detect α- tubulin expression. A rabbit anti-human γ-tubulin (1:400, Santa Cruz Biotechnology) antibody and 568-conjugated goat anti-rabbit IgG secondary antibody were used to detect γ- tubulin expression. The nucleus was stained with DAPI.

### Immunohistochemistry

Esophageal cancer tissues and animal tissues were embedded in paraffin and subjected to immunohistochemistry. Tissues were deparaffinized and hydrated and then permeabilized with 0.5% Triton X-100/1 × PBS for 10 min. Immunohistochemical staining for Ki-67 (1:100), phosphorylated (p)-histone H3 (1:100) or cleaved caspase 3 (1:200) was performed using the indirect avidin biotin-enhanced horseradish peroxidase method according to the manufacturer's instructions (Vector Laboratories, Burlingame, CA). After developing, all sections were observed by microscope (100×) and analyzed using Image-Pro Plus software (v. 6.2) program (Media Cybernetics).

### Molecular modeling

To study the binding and interaction of APIO-EE-9 with Aurora A or B, we performed *in silico* docking using the Schrödinger Suite 2015 [66]. First the Aurora A and B crystal structures were downloaded from the PDB Bank [67] and then prepared under the standard procedures of the Protein Preparation Wizard (Schrödinger Suite 2015). Hydrogen atoms were added consistent with a pH of 7 and all water molecules removed. The ATP-binding site-based receptor grids of Aurora A and B were generated for docking, respectively.

APIO-EE-9 was prepared for docking by default parameters using the LigPrep program. Then the docking of APIO-EE-9 with Aurora A and B, respectively, was accomplished using default parameters under the extra precision (XP) mode with the program Glide. By these methods, we obtained the best-docked representative structures.

### *In vitro* Aurora A and B kinase assays

The histone H3.3 protein (0.5 or 1 μg) was used as a substrate for Aurora A or B, respectively, and mixed with active Aurora A (100 ng) or B (50 ng) with different doses of APIO-EE-9 or hesperidin in a 20 μl (total volume) reaction. Respective mixtures were added to 1 mM ATP and kinase buffer (Cell Signaling Technology) at 30°C for 30 min for Aurora A or 5 min for Aurora B. Reactions were stopped by boiling the samples in 6X SDS buffer and proteins were analyzed by Western blot.

### *In vitro* ATP competitive binding and *ex vivo* pull-down assays

APIO-EE-9-conjugated Sepharose 4B beads were prepared following the manufacturer's protocol (GE Healthcare Biosciences). KYSE450 cell lysate (500 μg) or active kinase Aurora A or B (200 ng) was incubated with APIO-EE-9 Sepharose 4B beads or Sepharose 4B beads alone (negative control) at 4°C for 2 h. Then different concentrations of ATP were mixed with the respective beads and rocked at 4°C overnight and then analyzed by Western blot.

### Xenograft mouse model

Athymic nude mice (6-8 week) were obtained from Charles River and maintained under specific pathogen-free conditions. Mice were divided into 5 groups (n = 10 mice in each group). KYSE450 cells expressing shAurora A or shAurora B (1×10^7^/100 μl) were injected subcutaneously into the right flank of each mouse. Tumor volume was measured once weekly with calipers and volume was calculated using the formula, tumor volume = length× width× height× 0.52. All animal studies were performed following the guidelines approved by the University of Minnesota Institutional Animal Care and Use Committee.

### Patient-derived xenograft (PDX) mouse model

Esophageal cancer (1^st^ Affiliated Hospital of Zhengzhou University) fragments (2-3 mm) were implanted into immune deficient (SCID) mice. We generated enough mice for treatment with APIO-EE-9 in an *in vivo* PDX study. This study was approved under a protocol approved by the Zhengzhou University Institutional Animal Care and Use Committee (Zhengzhou, Henan, China). Mice were divided into 3 groups with 10 mice per group and included a vehicle control group and 2 APIO-EE-9-treated (40 or 200 mg/kg) groups. Four days after tumor implantation, mice were treated with vehicle control (0.9% saline) or APIO-EE-9. Body weight and tumor volume were measured once a week, tumor volume was calculated as described for the xenograft model above.

### Statistical analysis

Data are presented as mean values ± standard deviation (S.D.). Each experiment was repeated at least 3 times independently. Statistical significance was determined by one-way analysis of variance (ANOVA) and a statistically significant difference was defined as *p* < 0.05.

## SUPPLEMENTARY FIGURES


