# Supplementary material for: APIO-EE-9 is a novel Aurora A and B antagonist that suppresses esophageal cancer growth in a PDX mouse model
Source: Oncotarget. 2017 Jun 16;8(32):53387–404. doi: 10.18632/oncotarget.18508 (PMC5581118; doi:10.18632/oncotarget.18508)
Supplement: Supplementary file 1 [file oncotarget-08-53387-s001.pdf]

APIO-EE-9 is a novel Aurora A and B antagonist that suppresses esophageal cancer growth in a PDX mouse model

SUPPLEMENTARY MATERIALS

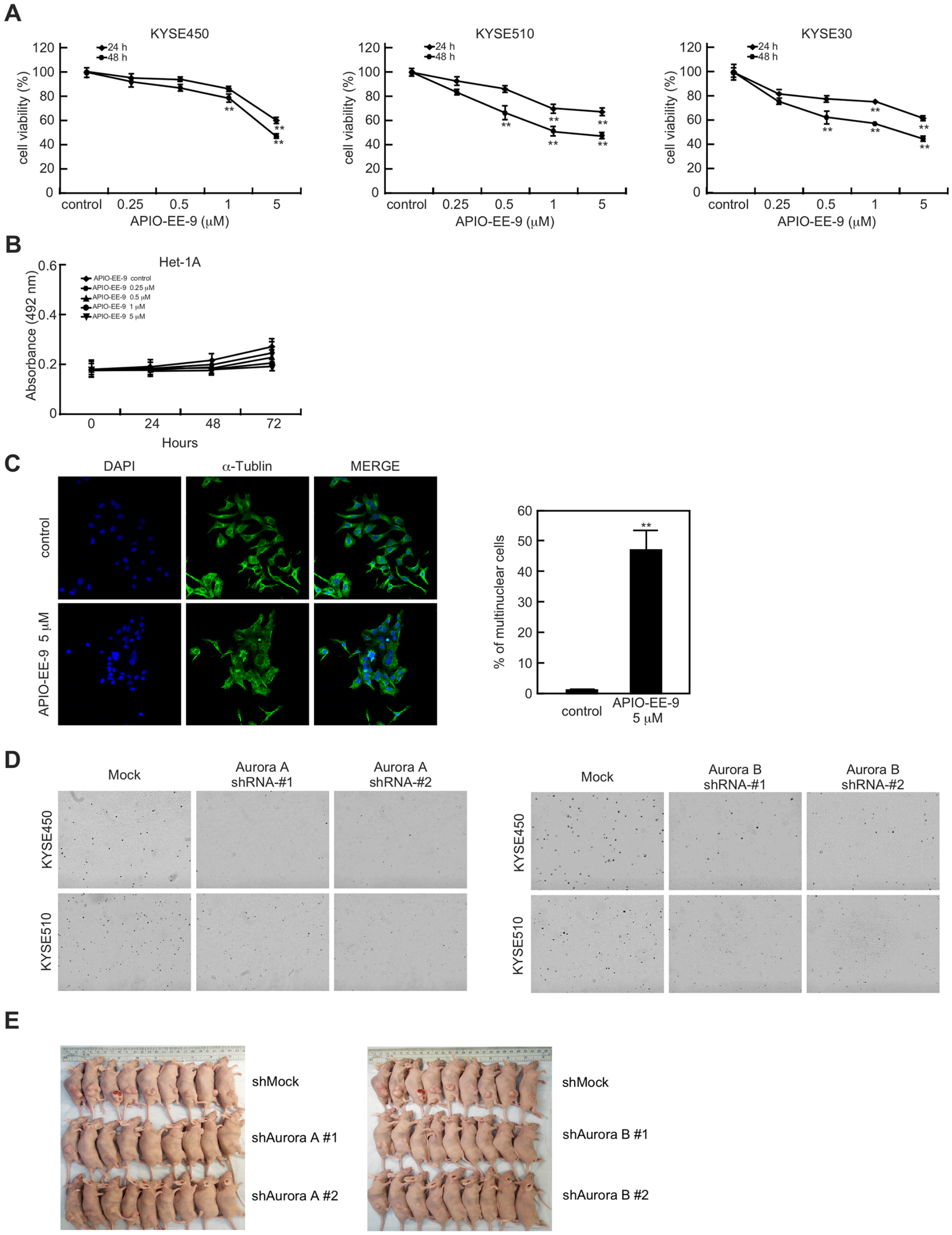

Supplementary Figure 1: Knocking down Aurora A or B expression in esophageal cancer cells inhibits anchorage-independent growth and tumor growth in a xenograft mouse model. **(A)** Cytotoxicity of APIO-EE-9 was measured by MTS assay in esophageal cancer cell lines. **(B)** The normal esophageal cancer cell line Het-1A was treated with different concentrations of APIO-EE-9 and viability was estimated. **(C)** APIO-EE-9 induced multinucleation in esophageal cancer cells. **(D)** Efficiency of Aurora A or B shRNA in KYSE450 and KYSE510 cells. Knocking down Aurora A or B expression in KYSE450 and KYSE510 cells inhibits colony formation. **(E)** Mice injected with knockdown cells were photographed.

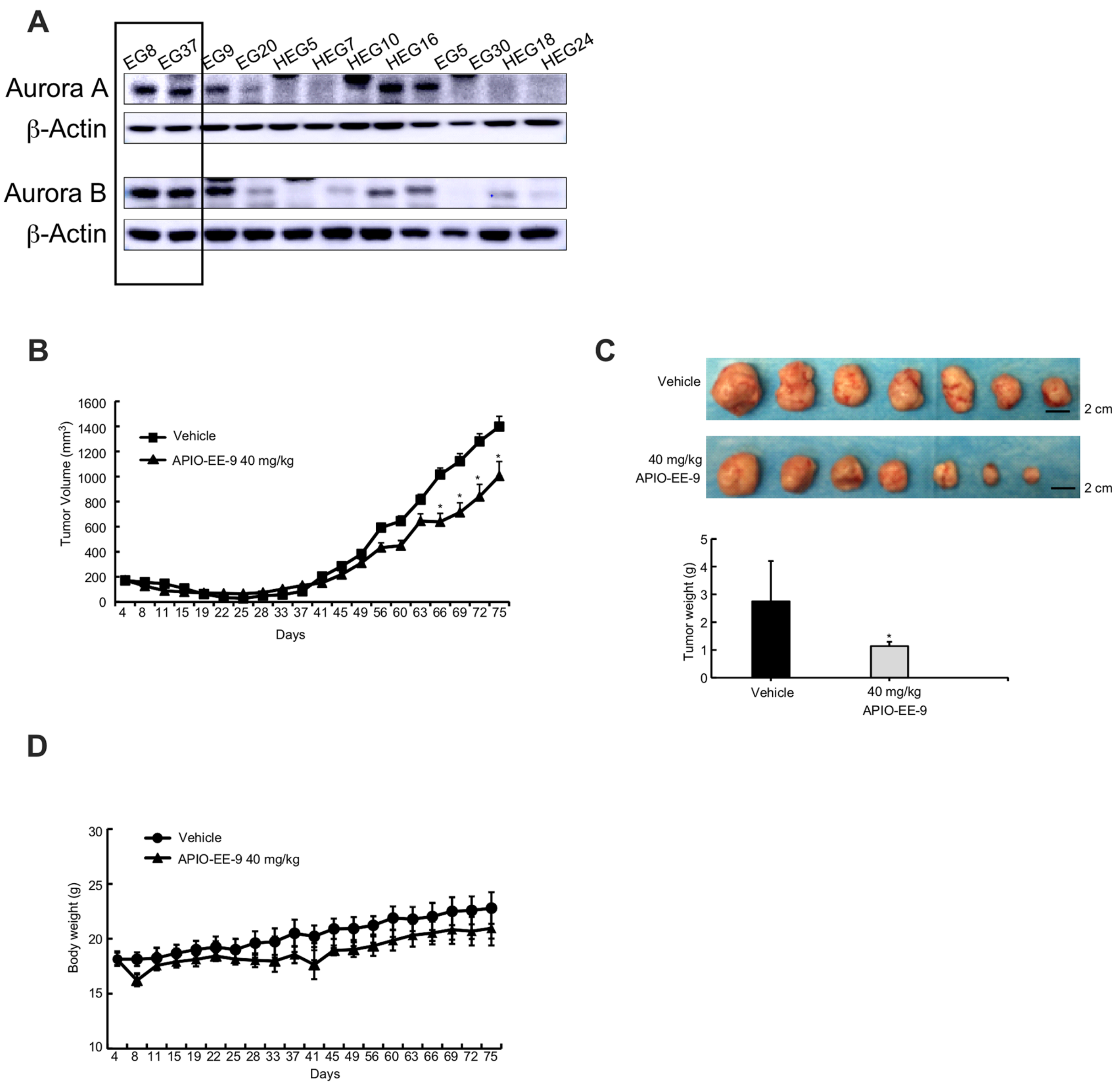

Supplementary Figure 2: APIO-EE-9 suppresses esophageal PDX tumor growth. **(A)** Patient tumors were harvested and Aurora A or B expression was detected by Western blot. **(B)** Treatment with APIO-EE-9 inhibits ESCC PDX tumor size compared with the untreated group. The asterisks (*) indicate a significant ($p < 0.05$) decrease in tumor volume in the APIO-EE-9-treated groups compared with the vehicle-treated group. **(C)** Mouse tumors were photographed (upper) and tumor weight determined (lower). The asterisk (*) indicates a significant ($p < 0.05$) decrease in tumors from mice treated with APIO-EE-9 compared with the vehicle-treated group. **(D)** Mouse body weight.
